# The Place of In Vitro Maturation in PCO/PCOS

**DOI:** 10.1155/2018/5750298

**Published:** 2018-07-31

**Authors:** Shital Julania, Melanie L. Walls, Roger Hart

**Affiliations:** ^1^King Edward Memorial Hospital, 374 Bagot Road, Subiaco, WA 6008, Australia; ^2^Fertility North, Suite 213, Specialist Medical Centre, Joondalup Health Campus, Shenton Avenue, Joondalup, WA 6027, Australia; ^3^Fertility Specialists of Western Australia, Bethesda Hospital, 25 Queenslea Drive, Claremont, Perth, WA 6010, Australia; ^4^Division of Obstetrics and Gynaecology, University of Western Australia, King Edward Memorial Hospital, 374 Bagot Road, Subiaco, Perth, WA 6008, Australia

## Abstract

In vitro maturation (IVM) of human oocytes is an emerging treatment option for women with polycystic ovary/polycystic ovary syndrome (PCO/PCOS) in addition to the standard in vitro fertilization (IVF) treatment. There has been significant improvements in pregnancy rates with IVM over the last two decades. This article reviews the place of IVM for women with PCO/PCOS, placing an emphasis on the predictors of successful pregnancy, optimization of culture media, IVM protocols, pregnancy rates, and neonatal outcomes following IVM treatment.

## 1. Introduction

Polycystic ovary syndrome (PCOS) is considered the most common endocrine disorder of women in their reproductive years and may lead to anovulation and infertility. It affects up to 4–12% of women generally [1, 2]. Various treatment modalities are used for treatment of PCOS-related infertility, including lifestyle modification as a first-line treatment for obese and overweight women with anovulation, ovulation induction with either oral agents or gonadotrophins and laparoscopic ovarian drilling as second-line therapy [1]. However, a subset of these patients will either be resistant to treatment or will fail to conceive despite ovulation induction treatment and will eventually need controlled ovarian stimulation (COS) and in vitro fertilization (IVF) [3]. Additionally, they may have compromised fallopian tube function or male factor infertility and require IVF from the start. However, when undergoing IVF treatment, women with PCOS are predisposed to developing ovarian hyperstimulation syndrome (OHSS) due to their high antral follicle count; this facet also make them ideal for in vitro maturation (IVM) treatment [3, 4]. OHSS is a significant cause of discomfort, distress, hospitalisation, and even mortality for women undergoing IVF treatment, due to the extravasation of fluid out of the vascular system leading to the development of ascites and potentially pleural effusion and thromboembolic phenomena [1, 5].

In vitro maturation of oocytes has been suggested as an alternative approach to conventional IVF as it completely avoids the risk of OHSS [6]. IVM treatment typically involves a relatively short duration of gonadotrophin stimulation and the retrieval of oocytes from follicles at a much smaller diameter than with conventional IVF treatment, often without the use of a trigger injection and oocyte maturation occurs in vitro [4]. The process of IVM involves the collection of immature oocytes at the germinal vesicle (GV) or metaphase I (MI) stages of meiosis, retrieved from small ovarian follicles, by transvaginal oocyte retrieval. Subsequently, these oocytes undergo resumption of meiosis and maturation to metaphase II (MII) oocytes in the laboratory.

The *in vivo* preparation for IVM treatment is a source of contention, and it has been suggested that cycles involving both gonadotrophin and an ovulation trigger should instead be referred to as “truncated” or “minimal stimulation” IVF [7] and not IVM, and the definition of true IVM has recently been debated in the literature by De Vos et al. [7]. By the administration of a human chorionic gonadotrophin (hCG) trigger prior to oocyte collection, “hCG priming,” the resumption of meiosis begins and subsequently oocytes are collected that may be at varying stages of the maturation process; GV, MI, or MII oocytes. In turn, this makes *in vitro* culture, fertilization, embryo culture timing, and embryo transfer logistically difficult, as the oocytes need to be treated individually according to their stage of development. In agreement with De Vos, it is our view that the true classification of IVM should be restricted to cycles without the use of a hCG trigger, with the process of germinal vesicle breakdown and resumption of meiosis completed “*in vitro*.” Hence, true IVM involves the culture of germinal vesicle (GV) oocyte in vitro culture.

## 2. History of IVM

The technique of IVM has been used in veterinary practice for a long time [8, 9]. However, the first pregnancy resulting from IVM in humans was reported in 1991 using donor oocytes from unstimulated ovaries from women undergoing gynaecological surgery [10]. In 1994, Trounson et al. reported a pregnancy in an anovulatory woman with PCOS after IVM of her own oocytes with an abbreviated steroid replacement protocol after embryo transfer [11]. Following these early reports, and likely due to the widespread uptake of ovarian stimulation, research progressed slowly for IVM. Initial reports focused on the development of specific culture conditions [12], variations in stimulation and priming protocols [13, 14], and patient selection [15, 16], as well as fertilization techniques [17]. Traditionally, cycles of IVM are performed using intracytoplasmic sperm injection (ICSI) for fertilization, although similar fertilization rates with IVM-IVF have been reported by Walls et al. making IVM-IVF an acceptable option, which is a cost-effective and potentially less invasive treatment than traditional IVF [18]. More recently, research has progressed to include assessments of IVM outcomes using the advanced technologies of time-lapse incubation [19] and preimplantation genetic screening [20]. Together with the introduction of freeze-all protocols to reduce the incidence of miscarriage and allow success rates closer to standard IVF [4], these advances have generated a renewed interest in IVM research, particularly for PCOS patients. Thus, we believe that despite the use of strategies to minimise the risk of OHSS, such as the use of gonadotrophin-releasing hormone (GnRH) antagonists for pituitary suppression [21], IVM should still be viewed as an alternative treatment option for women with PCOS.

## 3. Indications for IVM

The use of IVM for infertility treatment has several perceived advantages over conventional IVF for women with a high antral follicle count, such as women with PCOS. These include a shorter duration of stimulation and the use of less gonadotrophins. Additionally, there is the avoidance of the supraphysiologic levels of oestradiol, with its symptomatic benefits, and the opportunity to minimise exposure to high oestradiol concentrations for a woman undergoing ovarian stimulation for fertility preservation with breast cancer, or a woman with a thrombophilia, and the elimination of the risk of OHSS. However, the initial interest and enthusiasm for IVM has waned, due to the perceived lower pregnancy rates achieved with IVM treatment and the relatively recent introduction of easily accessible strategies to reduce the risk of OHSS. Such modifications in the stimulation protocols for women with PCOS, undergoing IVF treatment perceived to be at a significant risk of OHSS, include the use of GnRH antagonist protocols [22], with the use of a GnRH agonist as a trigger injection prior to oocyte retrieval, the concurrent use of metformin during stimulation [21, 23], and the use of dopamine agonists [5]. However, despite these strategies, OHSS still occurs, albeit with less frequency [3]. Further perceived benefits of an IVM treatment cycle include a lower treatment burden for the patient, a lower cost, greater patient safety, and an alternative to standard IVF treatment [4].

In addition, IVM can be used in patients with ovarian resistance to follicular-stimulating hormone (FSH) [24], fertility preservation of cancer patients (particularly women with leukemia and oestrogen-sensitive tumours), and endometriosis patients undergoing extensive endometrioma excision [24]. It can also be used as a fertility-preserving option for women at risk of premature ovarian failure [25]. It has also been used in normal responders with history of poor oocyte/embryo quality as well as for oocyte donation cycles to avoid the discomfort of the stimulation for a donor. Furthermore, the laboratory processes of IVM employed on immature oocytes derived from ovarian tissue enable clinicians to offer another option to preserve fertility for women who may be undergoing oophorectomy [26]. Segers et al. have reported a successful pregnancy after ex vivo method of oocyte cryopreservation after oophorectomy followed by IVM [27], and our group have performed oophorectomy after a few days of ovarian stimulation, without a trigger and we derived 18 mature oocytes after IVM [26].

Many couples drop out from IVF treatment due to the physical and psychological burden of conventional ovarian stimulation, and therefore, IVM can provide for some couples a less intense option that avoids the bloating discomfort of conventional treatment. Furthermore, in countries where the patient is required to pay for her medication, IVM offers a shorter, minimal stimulation approach at a lower cost. In addition, IVM may be used for patients who live in a rural or remote environment who are at risk of OHSS after COS, requiring intense post egg collection monitoring and risk a cycle cancellation where the requirement for frequent observation poses logistical problems, such as in our rural environment in Western Australia.

## 4. Improving the Success of IVM

The major reason why IVM has not been adopted more widely to treat women with PCO or PCOS is due to the perceived reduced likelihood of successful treatment. Hence, this led to the adoption of treatment protocols involving the transfer of multiple embryos in a fresh treatment cycle [28]. Earlier studies that compared the outcomes of IVM to conventional IVF reported significantly worse pregnancy rates with IVM, as the majority of these treatment protocols involved hCG priming. This led to the early resumption of meiosis, and due to the short duration of the follicular phase of all IVM protocols, a poor luteal phase endometrium developed. Hence, the adoption of treatment protocols using a short period of ovarian stimulation, the avoidance of hCG priming, and the adoption of a “freeze-all” [6, 29] approach with the transfer of a single blastocyst in a subsequent frozen embryo transfer cycle have led to live birth rates that approximate those of traditional IVF cycles, with the avoidance of OHSS. Good patient selection, optimization of IVM protocols, oocyte retrieval procedure, and potentially improving culture media may offer future potential to improve treatment outcomes.

## 5. Optimization of IVM Protocol

Various IVM protocols have been described, with oocyte aspiration performed in unstimulated cycles or stimulated cycles with FSH priming and with or without HCG trigger [6, 30–32]. Although success rates were low in initial IVM studies, with improved regimes and protocols, the rates of oocyte maturation, fertilization, and implantation have been significantly improved [4, 6].

The effect of various IVM protocols using no priming, FSH only, hCG only, and FSH with hCG, had been studied by Fadini et al. in normoovulatory women [31] and reviewed by Siristatidis et al. [33]. Their data demonstrates the use of FSH with hCG improved clinical pregnancy rates and implantation rates in a randomized trial [31]. The effects of FSH priming in the follicular phase are due to the recruitment of greater number of follicles, whereas hCG priming causes maturation of some follicles in vivo leading to recruitment of oocytes at different stages [6, 32]. Hence, in IVM cycles with hCG priming, it is possible to collect oocytes in various stages of maturity from follicles from 2–13 mm in size [14, 34]. In a sibling oocyte study, Son et al. reported that after hCG priming, the embryo development was similar irrespective of the size of the follicle the oocyte was aspirated from, whether larger or smaller than 10 mm in diameter [35]. Hence, it would appear that the timing of oocyte retrieval is not so critical when hCG priming is used; however, it is critical when no trigger is used. Both our group and the Belgian group (De Vos et al. and Ortega-Hrepich et al.) have found improved clinical outcomes with transfer of single vitrified-warmed embryos in non-hCG-primed IVM cycle in PCOS patients, as compared to fresh embryo transfer [6, 29]. These effects are mainly attributed to poor endometrial receptivity in fresh embryo transfer cycles. With the opportunity to introduce adjuvants to the culture media such as C-Type natriuretic peptide (CNP) and amphiregulin, the optimal follicular size at the time of retrieval in non-hCG-primed cycles may reduce to 8 mm [36].

With regard to the follicle aspiration technique employed in an IVM cycle, most centres use a small gauge needle (16 or 17 gauge) with suction pressures ranging from 52 mm to 200 mm Hg, with either a single or double lumen needle; in our unit, we use a double lumen needle to enable follicular flushing [6, 37–42]. When Junk and Yeap published their optimized IVM protocol from our clinic in 2012 by the use of IVM in combination with FSH priming, the collection of oocytes when the leading follicle was 10–12 mm in diameter and the transfer of a single blastocyst-stage embryo with modified hormone therapy to assist endometrial development, they demonstrated excellent implantation and pregnancy rates [6]. With ongoing evaluation of our IVM cycle results, we now just perform an embryo transfer in a subsequent vitrified-warmed cycle, as our clinical pregnancy rates are the same as our IVF cycle results for women with PCOS [4].

Many studies have described excellent pregnancy rates using FSH or/and hCG priming [4, 6, 31]. A Cochrane review reported that hCG priming for IVM treatment had no effect on pregnancy, live birth, or miscarriage rates; however, the evidence was low, due to the limited amount of studies available for review [43]. Regardless, this evidence, coupled with the logistical difficulties encountered following hCG priming and the more recently reported success rates following IVM treatment without hCG priming, demonstrates that hCG priming is not an advisable methodology in IVM treatment.

## 6. Predictive Markers of Success of IVM

A recent study by Tannus et al. found that the most significant predictors for live birth after IVM in PCOS patients are a short duration of infertility, a higher oocyte retrieval number, a higher number of blastomeres within the embryo, and a better embryo grade. Potentially, these predictive factors can be used when planning treatment or counselling patients [3]. In addition, the paper by Walls et al. demonstrated very poor IVM treatment outcomes for women over 36 years of age [4].

The serum anti-Mullerian hormone (AMH) concentration and the antral follicle count (AFC) are useful factors for the prediction of pregnancy outcomes for women with PCOS prior to the commencement of an IVM cycle [44, 45]. Seok *et al.* reported in a retrospective case-control study of patients with PCOS that women with serum AMH concentrations above 8.5 ng/mL had IVM pregnancy outcomes comparable to women undergoing conventional IVF treatment [44]. Furthermore, the serum AMH and the AFC appear to be independent predictors of cumulus oocyte complex (COC) yield, with the cumulative, ongoing clinical pregnancy rate being greater for women who had more than eight COC retrieved. Guzman et al. described a predictive model of IVM success incorporating the serum AMH and AFC [45]. As would be expected, the presence of an abundance of antral follicles, which predispose a woman with PCOS susceptible to OHSS when undergoing IVF treatment, in fact makes IVM treatment ideal for such women.

However, the pregnancy rates in unstimulated hCG-primed IVM cycles appear to be impaired in women with PCOS with insulin resistance, as hyperinsulinemia appears to have a negative effect on endometrial function and the implantation process rather than embryo quality [46]. In addition, the ratio of the serum gonadotrophins has reportedly had no difference on pregnancy rates in woman with PCOS undergoing IVF with GnRH agonist, GnRH antagonist, and IVM cycles [47].

## 7. Optimization of Culture Media

Until recently, maturation media formulations and culture protocols did not differ significantly from one another, except for more than 24-hour variations in culture timing (generally reported between 24 h and 48 h) and occasional variation in culture media additives. At a basic level, IVM culture media consists of a base culture media, hormonal additives, and a source of protein. Reported base media consist of either commercially available IVM media [48] or blastocyst defined media [6] with no reported differences in success rates between the two [49]. For successful resumption of meiosis, the addition of either FSH and either hCG or LH to the culture media is necessary to promote the proliferation and expansion of the coronal cells and aid in the final stages of oocyte maturation in vitro. Interestingly, one study demonstrated that after oocyte retrieval without hCG priming, the larger GV oocytes have the greater potential for meiotic resumption [50]. Most clinical protocols reported have included either autologous maternal serum, human serum albumin (HSA), or human follicular fluid (HFF) as a source of protein for use in culture with comparable efficacy [51]. Preference may be given to HSA, as HFF and maternal serum have the potential to introduce contaminants and other elements which may impact negatively on oocyte or embryo developmental competence, as well as contributing to the lack of heterogeneity across cases, as they do not allow for adequate quality control.

Other culture additives have been suggested to improve IVM success rates over the years; however, their reports are sporadic and rarely used in everyday culture. Insulin-like growth factor (IGF-I) has shown promise in animal models and early human studies, promoting cumulus cell expansion [52], and recombinant epidermal growth factor has been added with success to some culture systems [53] as well as its family members amphiregulin and epiregulin showing promise in terms of maturation rates and embryo developmental capacity to the day two to three stage [54]. In recent years, the discovery of other factors which may promote oocyte maturation such as oocyte-secreted factors BMP-15 and GDF9 and their heterodimer “cumulin” has shown promise in animal models [55], and we have seen the emergence of dynamic *in vitro* systems to improve embryo quality and quantity, the so-called prematuration or pre-IVM systems [56]. One of the important aspects is to maintain optimal concentration of cyclic adenosine monophosphate/cyclic guanosine monophosphate (cAMP/cGMP) levels after removal from the follicle, as they play an important role in oocyte meiosis resumption/arrest [56]. Pre-IVM with cAMP modulators have been shown to improve IVM outcomes in bovine oocytes [57], and a recent study demonstrated a strategy involving prematuration culture (PMC) in the presence of CNP followed by IVM using FSH and amphiregulin, which increased oocyte maturation potential, leading to a higher availability of day three embryos and good-quality blastocysts for single embryo transfer [36]. Like most research in the IVM field, this will need to be validated by further large-scale trials.

## 8. Safety of IVM

One of the primary concerns regarding IVM treatment are the neonatal outcomes and any adverse effects on the growth and development of children born following the procedure. Increased rates of congenital malformations have been reported in children born following conventional IVF treatment compared with the general population [58], as well as a potential increase in metabolic disorders [59]. While there is currently limited evidence of the long-term outcomes of children born following IVM, early research has demonstrated that outcomes are comparable to conventional IVF controls [60].

With respect to embryonic development, our group have reported an increase in early embryo arrest in women with PCOS after IVM as compared to women with PCOS undergoing standard ICSI using time-lapse analysis, although no difference was recorded in the morphokinetic development of the useable embryos between the groups [19]. We have also noted that PCOS-IVM oocytes were significantly larger as compared to the oocytes of women from PCOS-ICSI and control-ICSI groups [61]. These differences had been attributed to the in vitro maturation process with inadequate completion through the stages of cytoplasmic maturation. These changes may be associated with a decrease in the rate of fertilization and impaired blastocyst development for PCOS patients undergoing IVM. A similar finding was recorded in a recent study by Roesner et al. using time-lapse analysis, where significant differences were noted in embryo development between PCOS-IVM as compared to PCOS-ICSI and control-ICSI groups, with similar pregnancy and live birth rates resulting in these groups [62]. The rates of embryo development differed between these two studies, and this is attributed to the difference in IVM protocols used (e.g., FSH priming or FSH and hCG priming, or potentially the duration of FSH use), differences in IVM culture media, or possibly patient demographics.

There have been concerns regarding the association of epigenetic defects with IVM treatment. Recent gene studies have shown reassuring results, although the small sample size is a limiting factor of these studies. Pliushch et al. studied 15 developmentally important genes and two repetitive elements for methylation levels in 11 patients undergoing IVM treatment and 19 patients undergoing standard IVF/ICSI. They analysed tissues from chorionic villous sampling and cord blood sampling and demonstrated minimal effects of IVM treatment on the methylation patterns of the sampled tissue [63]. Using the same technique of bisulphite pyrosequencing for analysis of gene methylation patterns, Kuhtz et al. studied three maternally methylated and one paternally methylated gene for imprinting errors and found no differences in the methylation patterns in these genes after IVM treatment as compared to in vivo-developed oocytes [64]. Thus, these studies provide some reassuring data regarding any potential epigenetic effects resulting from IVM treatment.

Junk and Yeap reported no congenital defects in 28 patients who had live birth in their study [6]. In the review of IVM strategies by Mikkelsen in 2005, of the 46 patients who delivered a baby, none of the children conceived after IVM had chromosomal abnormalities, one baby had a soft cleft palate, and there was one stillbirth that was not attributable to IVM [30].

With regard to the obstetric outcomes after IVM treatment, the preterm birth rates and the infant birth weights, both important predictors of health outcomes, are comparable after IVM and standard IVF conception, with a possible lower preterm birth rate after IVM treatment [4]. In a French study, the authors reported two-year follow-up of children born after IVM treatment in comparison to those born after standard ICSI treatment. In their study, the mean weight and height of boys were similar amongst the two groups, although girls were significantly heavier in the IVM group [65]. Fadini et al. also reported higher birth weight in singleton children born after IVM [66]. The mean birth weight in IVM infants was higher than spontaneously conceived infants, potentially due to the higher risk of gestational diabetes in women with PCOS. Another study reported that in comparison to the general population, the mean gestational age at delivery and birth weight, for both singletons and twins, was comparable to the general population [67]. However, as concerns have been raised regarding the possibility of epigenetic changes resulting from IVM treatment, larger studies are required [68].

With regard to childhood development, a recent prospective controlled study comparing the embryonic, neonatal, and two-year developmental outcomes in children born after IVM, IVF, and ICSI treatments demonstrated no difference in Bayley' developmental scores between the groups [60]. In another two-year follow-up study of children born from IVM treatment, recording the growth and development using Bayley's scales, the authors reported normal scores for 34 out of 35 children and a mild development delay in one child. Their neuropsychological scores at two years of age were normal in this study. Furthermore, in another study, a cohort of children born after IVM in women with PCOS patients matched with spontaneously conceived children, when they underwent developmental assessment between 6 and 24 months of age using Bayley's scales, there were no differences in their mental or psychomotor development and no concerns regarding their neonatal or early infancy development [69]. Thus, the outcomes of IVM have been reassuring so far; however, the sample size in these studies is small, warranting interpretation of results with caution and emphasizing the need for further study.

## 9. Conclusions

Tannus et al. have reported clinical pregnancy rates of 44.7% and live birth rate of 34.6%, for women undergoing IVM treatment, with the majority of transfers being single [3]. Furthermore, our group compared the cumulative live births obtained after IVM treatment and conventional IVF/ICSI treatment, for patients with PCOS in Western Australia, and reported similar per frozen embryo transfer cycle pregnancy rates across both groups. However, we recorded a higher cumulative live birth rate achieved after standard IVF in comparison to IVM treatment (55% versus 41%) [4]. The authors attribute this finding to the lower number of MII oocytes obtained in the IVM group in comparison to the IVF/ICSI group, where roughly half the number of oocytes are retrieved as follicles are aspirated at an IVM collection. Importantly, embryo development per MII oocyte was similar, and the embryo implantation potential was also similar when examined in freeze-thaw cycles [4]. Consequently, the improvement of the MII oocyte rate is the key to further optimize the potential of IVM as a technique. Importantly, there were no cases of OHSS in the IVM group, whereas seven patients in the IVF/ICSI group developed OHSS; consequently, the elimination of OHSS is a significant advantage of IVM making it a safer option and potentially a more “patient friendly” approach. There were no multiple pregnancy or births in the IVM group and only two sets of twins in the IVF group, attributed to the predominantly single blastocyst transfer approach [4].

The adoption of a “freeze-all” approach has led to the avoidance of the difficulty in overcoming the poor luteal phase in a fresh IVM cycle and has been adopted now as routine in our practice [4]. Also, other groups have demonstrated that the implementation of hormone therapy regimens including high-dose oestrogen therapy commenced earlier in the treatment cycle may lead to an improved endometrial environment for embryo implantation, in comparison to other regimes [6].

A recent meta-analysis of IVM protocols, with and without the use of FSH and with and without hCG priming, has provided evidence demonstrating that IVM seems to be the preferable approach in treating women with PCOS during an IVF cycle as compared to those without PCOS [33]. This meta-analysis included 11 trials with 268 PCOS, 100 PCO patients, and 440 women with other causes of subfertility; they concluded that IVM appears to be a more efficient treatment option in terms of clinical pregnancy, implantation and cycle cancellation rates for women with PCOS when compared to the non-PCOS group. They also observed a borderline, but meaningful, trend in live birth rates in the PCOS group, favouring IVM [33]. Oocyte maturation and miscarriage rates did not differ between the groups, while a borderline trend towards lower fertilization rates among PCOS patients was observed. Previously, the same group in 2013 were unable to find any randomized control trials with the intention to perform IVM before IVF or ICSI in PCOS patients. They state that it is imperative that large multicentre studies are required in the field of IVM to answer the question whether IVM should be done prior to standard IVF/ICSI in PCOS women [70]. However, before such a study were to commence, a standardized IVM protocol must be agreed upon; with or without the use of FSH stimulation, either with or and without hCG priming, and whether to include a fresh or just the frozen transfers of a single embryo.

The IVM approach offers an excellent treatment option for women with PCOS, who are required to undergo assisted reproduction, as many subfertile women with PCOS will conceive with ovulation induction therapy alone. IVM offers several advantages over standard IVF, particularly the elimination of the risk of OHSS, it is cheaper and with a lower side effect profile than IVF, and offers a “patient friendly” approach to assisted reproduction.
